# A Rare Case of Uncorrected Tetralogy of Fallot With Right Hemitruncus in an Adult Survivor Highlighting the Role of Cardiac Computed Tomography

**DOI:** 10.7759/cureus.115233

**Published:** 2026-08-26

**Authors:** Jyoti Choudhary, Ami V Patel, Chetana Ratnaparkhi, Avinash Dhok, Kumar Rajnish Anand

**Affiliations:** 1 Department of Radiodiagnosis and Interventional Radiology, All India Institute of Medical Sciences, Nagpur, Nagpur, IND

**Keywords:** anomalous pulmonary artery, cardiac ct, case report, hemitruncus, tetralogy of fallot

## Abstract

Tetralogy of Fallot (TOF) is one of the most common congenital cardiac anomalies. The association of TOF with anomalous aortic origin of pulmonary artery (Hemitruncus) is, however, exceedingly rare. Survival into adulthood without surgical correction is itself unusual and typically depends on compensatory Major Aortopulmonary Collateral Arteries (MAPCAs). We present a case of a 30-year-old man who presented with recurrent hemoptysis and cough. Electrocardiogram (ECG)-gated cardiac computed tomography (CT) revealed the classical features of TOF, along with an atretic main pulmonary artery, right Hemitruncus, and reformation of the left pulmonary artery by multiple MAPCAs. This case illustrates the role of Cardiac CT for this exceptionally rare congenital heart anomaly in an adult survivor for anatomy delineation and treatment planning in this setting, which poses a management challenge.

## Introduction

Tetralogy of Fallot (TOF) is one of the most common cyanotic congenital heart diseases (CHD). In uncorrected cases, survival beyond adulthood is uncommon and depends on the presence of Major Aortopulmonary Collateral Arteries (MAPCAs) [1]. Classic autopsy-based data indicate that only 66% of patients with unoperated TOF survive to 1 year of age, 49% to 3 years, and 24% to 10 years, and survival is significantly worse when pulmonary atresia rather than stenosis is present [2]. The outcome depends critically on the number, size, and distribution of MAPCAs [2].

Anomalous origin of a branch pulmonary artery from the Ascending Aorta (AAo), i.e., Hemitruncus, accounts for < 0.1% of CHDs [3,4]. Its association with TOF is exceptionally rare (~0.4%) [5]. Normal cardiac development involves division of the truncus arteriosus by the aortopulmonary septum into the ascending aorta and main pulmonary artery, with the left and right pulmonary arteries arising from the sixth aortic arches. In Hemitruncus, there is abnormal migration or malalignment of this septum that results in entrapment of one pulmonary artery on the aortic side of the septum. Left-Hemitruncus arises from failure of the left sixth aortic arch to develop, preventing the origin of the left pulmonary artery from the main pulmonary artery and subsequently leading to its origin from the aorta. On the other hand, right-Hemitruncus occurs when there is incomplete migration of the right sixth aortic arch. This will lead to lifelong exposure of the affected lung to systemic arterial pressures, with major volume overload [3-6], predisposing that lung specifically to pulmonary vascular obstructive disease and Eisenmenger physiology.

Pre-treatment assessment of these anomalies is a cornerstone of imaging, and electrocardiogram (ECG)-gated cardiac computed tomography (CT) is the imaging modality of choice in such complex situations [7]. We report a rare case of TOF with Hemitruncus, diagnosed on cardiac CT.

## Case presentation

A 30-year-old man with a known uncorrected case of TOF presented with complaints of recurrent hemoptysis and cough for five days without fever or dyspnea (New York Heart Association (NYHA) Class I). There was a past history of multiple hospital admissions for cyanosis. There was no history of loss of appetite, weight loss, or significant family history. On examination, oxygen saturation was 76% on room air. Cardiovascular examination demonstrated a right ventricular heave, a single loud second heart sound, an ejection systolic murmur at the left upper sternal border, and a continuous murmur over the right hemithorax. Digital clubbing was present. The laboratory parameters are illustrated in Table 1.

**Table 1:** Laboratory parameters of the patient Laboratory parameters of the patient demonstrates polycythemia with a deranged coagulation profile.

Parameter	Result	Reference Values
Hemoglobin	23.1 g/dL (increased)	13-17 g/dl
Prothrombin Time (PT)	25.7 seconds (prolonged)	14.09-16.33 seconds
International Normalized Ratio (INR)	1.95	
Activated Partial Thromboplastin Time (aPTT)	39.8 seconds (prolonged)	28.04 - 37.83
D-dimer	0.1 μg/mL	<0.50 μg/mL
Sputum for Acid-Fast Bacilli (AFB)	Negative (three consecutive samples)	-
Sputum Cytology	Negative for malignant cells	-

**Figure 1:**
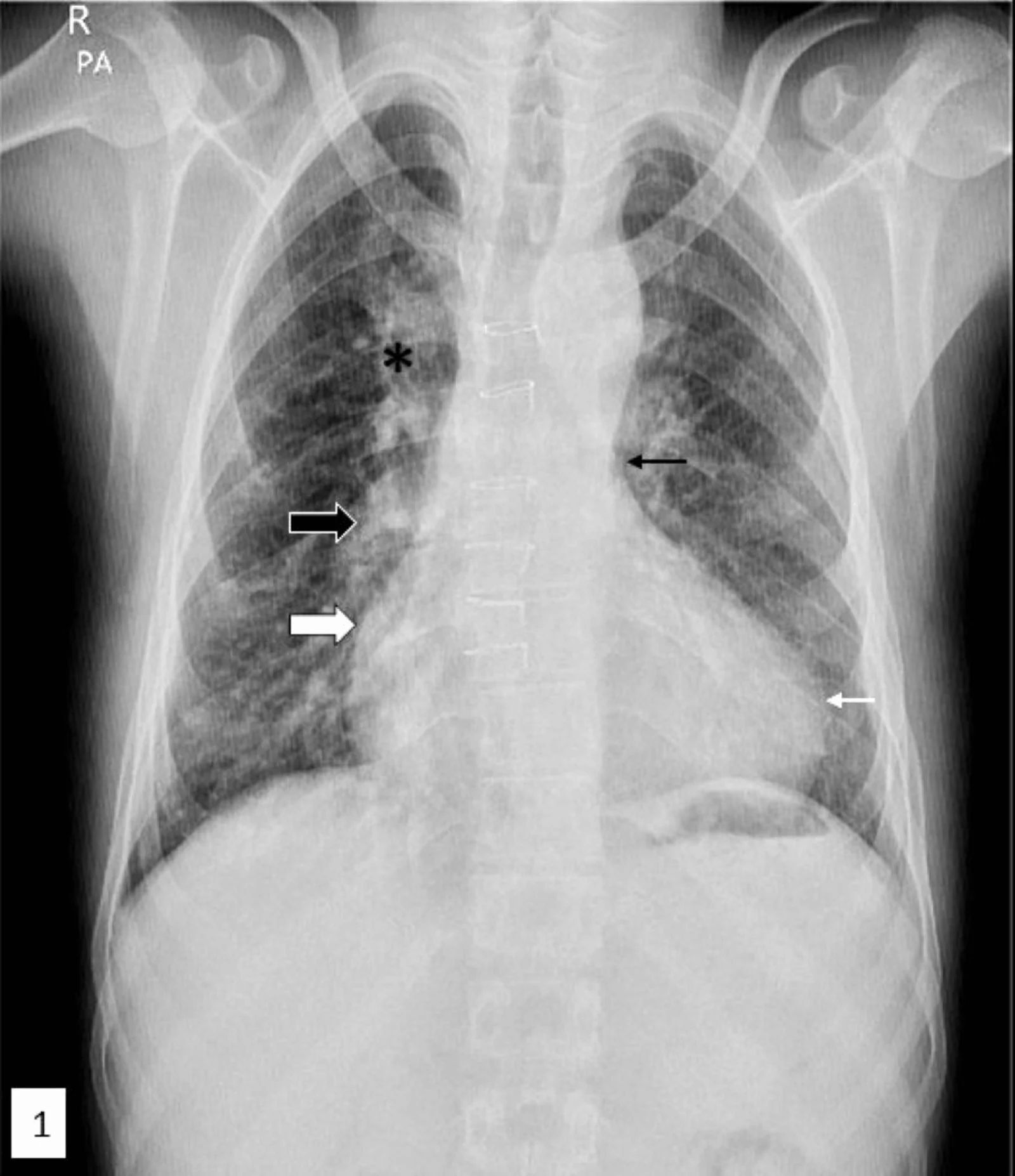
Chest radiograph of the patient Posteroanterior chest radiograph showing mild cardiomegaly with prominence of the right cardiac border (white block arrow) and slightly upturned cardiac apex (white arrow). There is marked concavity at the level of the pulmonary artery segment along the left heart border with loss of the normal 2nd mogul (black arrow). There is increased lung markings in the right lung (asterisk) with a prominent right descending pulmonary artery (black block arrow).

Posteroanterior chest radiography demonstrated mild cardiomegaly, concavity of the aortopulmonary window, reduced vascularity in the left lung, and increased broncho-vascular markings in the right lung (Figure 1).

The patient underwent Echocardiography, which demonstrated a large subaortic ventricular septal defect (VSD) measuring 14 mm with R->L shunt, overriding of the aorta, and severe right infundibular and valvular pulmonary stenosis favoring TOF morphology. The Main Pulmonary Artery (MPA) was not well visualized, suggestive of atresia. Left ventricular systolic function was preserved with an ejection fraction of approximately 55-60%.

The patient was advised cardiac CT evaluation. ECG-gated cardiac CT was performed on a 256-slice dual-source Siemens Somatom Drive following intravenous injection of 80 ml of Iohexol at 350 mg iodine/ml at a flow rate of 5 ml/s using a bolus-tracking technique. 3D Volume Rendering Technique (VRT) and Multiplanar Reconstruction (MPR) post-processing were performed in syngo.via software (Siemens Healthcare Limited, Erlangen, Germany).

**Figure 2:**
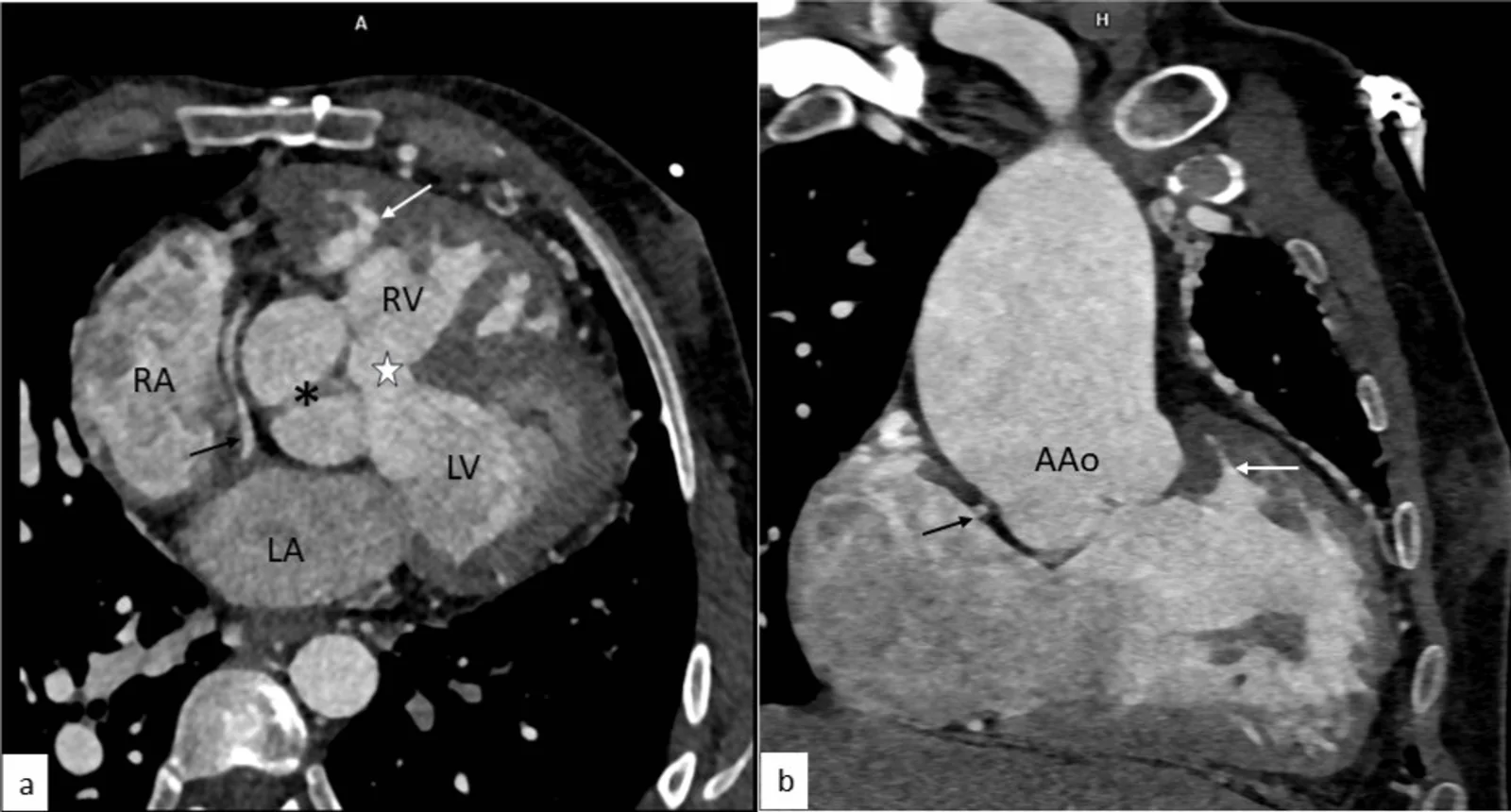
Cardiac CT demonstrating TOF morphology (a) Multislice Cardiac CT: (a) Axial 4- chamber view in diastole, showing subaortic VSD (star) with overriding of the aortic root (asterisk), right ventricular hypertrophy (white arrow) with crowding of papillary muscles. The RA appears dilated. There is a malignant course of the right coronary artery (RCA) between the RA and the aortic root (black arrow); (b) Multislice Cardiac CT: (b) Coronal image focusing on ventricular outflow tract showing marked infundibular stenosis (white arrow) and pulmonary atresia and overriding of the ascending aorta (AAo). Note is made of the RCA between the RA and the aortic root (black arrow). TOF-Tetralogy of Fallot, AAo-Ascending aorta, RA-Right atrium, RV- Right ventricle, LA-Left atrium, LV-Left ventricle, VSD-Ventricular septal defect.

CT revealed situs solitus with levocardia. A large subaortic VSD measuring approximately 21 mm with 50% aortic override was identified. Severe infundibular narrowing and right ventricular outflow tract (RVOT) obstruction were present, with a residual lumen of approximately 4 mm, along with mild right ventricular hypertrophy (Figures 2a, 2b).

**Figure 3:**
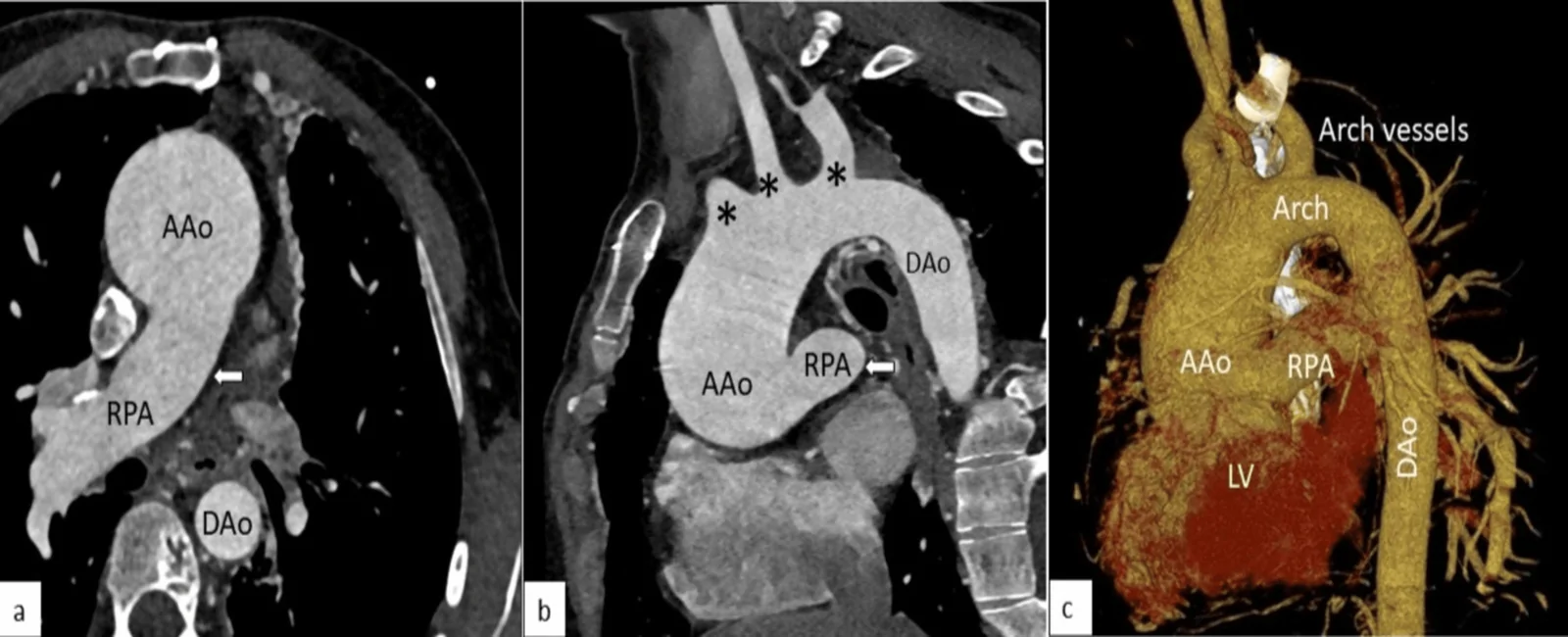
Cardiac CT showing right Hemitruncus (a and b) Multislice Cardiac CT (a) Axial and (b) Sagittal (reformatted oblique) images showing anomalous origin of RPA from ascending aorta (white arrow); (b) Sagittal (reformatted oblique) images showing anomalous origin of RPA from ascending aorta (white arrow) with normal origin of the arch vessels (asterisks). (c) Volumetric reconstruction image showing the origin of the RPA from the Ascending aorta. RPA- right pulmonary artery, AAo- Ascending Aorta, DAo- Descending aorta, LV- Left ventricle.

The Right Pulmonary Artery (RPA), measuring 25 mm, was seen arising from the posterolateral AAo approximately 20 mm above the sino-tubular junction and coursed posteriorly to the right hilum, consistent with right Hemitruncus. The right descending pulmonary artery measures 19 mm and appears prominent. No evidence of peripheral pruning of pulmonary vasculature was seen. The AAo was mildly dilated, measuring 50 mm in caliber. Left-sided aortic arch was seen with normal branching vessels (Figures 3a-3c).

**Figure 4:**
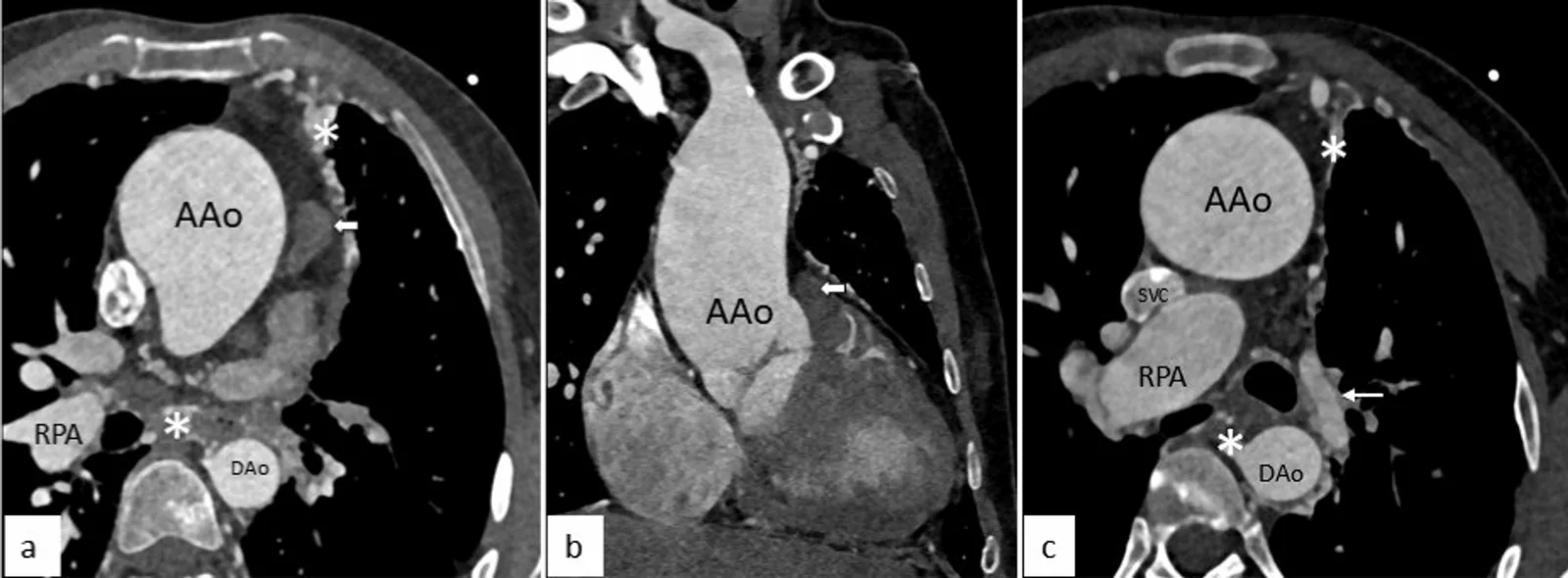
Cardiac CT demonstrating atretic MPA with reformed LPA (a) Multislice Cardiac CT Axial and (b) coronal image showing non-opacified atretic MPA (block arrow). Multiple mediastinal and peribronchial MAPCAs are seen(asterisk), (c) Cardiac CT Axial image, arrow showing reformed small caliber left pulmonary artery (LPA) in left hilum (white arrow) via multiple MAPCAs (stars). RPA- right pulmonary artery, AAo- Ascending Aorta, DAo- Descending Aorta, MPA- Main pulmonary artery, SVC- Superior Vena Cava.

The MPA measured only 3 mm and lacked a discernible pulmonary valve, suggesting atresia. The Left Pulmonary Artery (LPA), measuring 6.6 mm, was reconstituted at the hilum entirely through multiple MAPCAs originating from the descending thoracic aorta and bronchial arteries (Figures 4a-4c).

**Figure 5:**
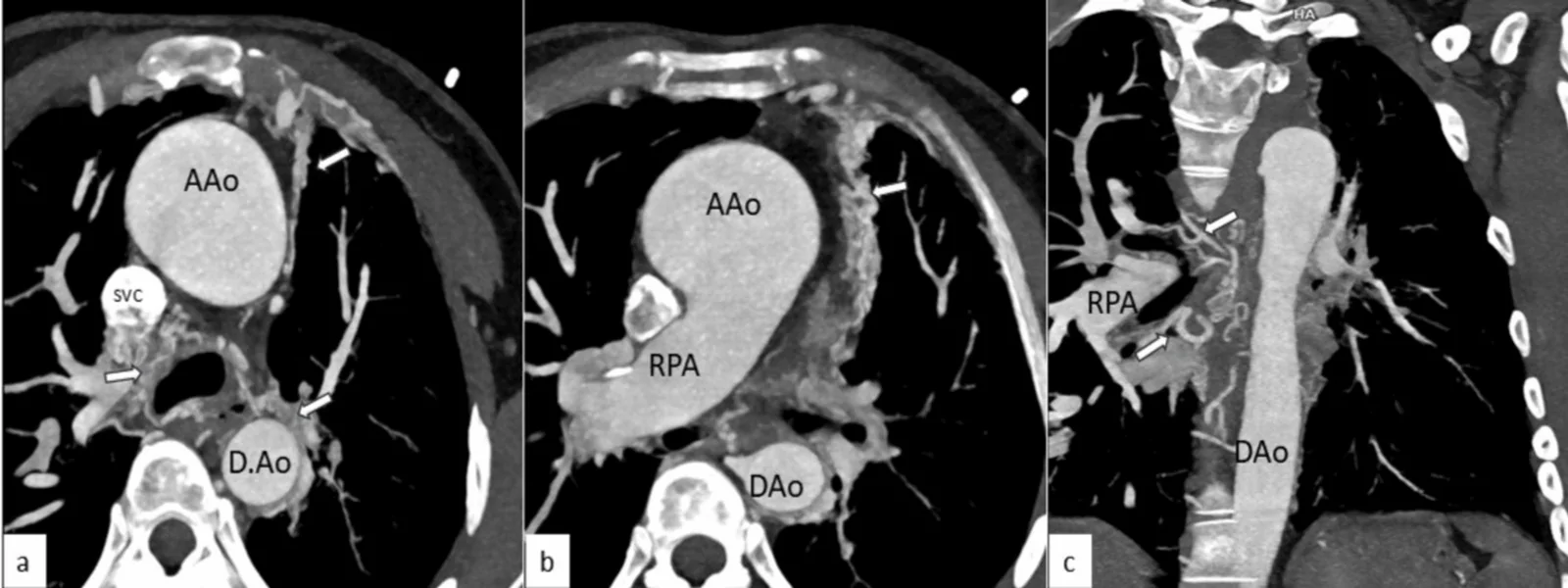
Cardiac CT demonstrating extensive mediastinal collaterals (a to c) Cardiac CT Axial images showing multiple extensive collaterals in the subcarinal, AP window, peribronchial, along the tracheal wall, mediastinal, bilateral hilar, and epicardial surface (block arrows). RPA- right pulmonary artery, AAo- Ascending Aorta, DAo- Descending Aorta, RPA- Right pulmonary artery, SVC- Superior vena cava.

Multiple MAPCAs and extensive smaller collaterals were seen in the mediastinum in the paratracheal, paraoesophageal, prevertebral, bilateral hilar, prevascular, paraaortic, subcarinal, and peribronchial regions along the walls of mainstem bronchi, along the pericardium, left cardiophrenic recess, costal pleura on the anterior aspect of the left side, and the perigastric region (Figures 5a-5c).

Based on the Cardiac CT, the final diagnosis is Uncorrected TOF with pulmonary atresia, right Hemitruncus, and MAPCA-dependent reformation of the LPA. The definitive management would require cardiac catheterization and vasoreactive testing to differentiate between the elevated pulmonary vascular resistance and irreversible Eisenmenger physiology; however, the patient opted out of any invasive procedure. 

Hemoptysis was resolved with conservative treatment, and no recurrence was observed during follow-up at one month and three months. Medical management was continued with 40 mg of propranolol twice a day, and selective embolization was advised if recurrent hemoptysis developed.

## Discussion

The coexistence of TOF, right Hemitruncus, and exclusive MAPCA-dependent contralateral pulmonary circulation in an adult survivor is exceptional [5]. Patel et al. described an adult patient with unrepaired TOF and right Hemitruncus diagnosed using echocardiography and catheter angiography [5]. In contrast, the present case was diagnosed on cardiac CT, which offers non-invasive, high-resolution evaluation with excellent depiction of the underlying anatomic abnormality and comprehensive mapping of MAPCAs, while avoiding the added procedural time and risk of catheter angiography, highlighting its current diagnostic role [6,7]. Talwar et al., in the largest series of Hemitruncus associated with TOF (n=17), reported four treated patients; none demonstrated exclusive MAPCA-dependent pulmonary supply or survived till adulthood [6]. 

Survival depends on an intricate hemodynamic balance that differs between the two lungs in this patient. The right lung, supplied by the anomalous RPA, is exposed to systemic pressure from birth. It is therefore at greatest risk of developing pulmonary vascular obstructive disease and Eisenmenger physiology, which is clinically significant because Eisenmenger syndrome generally precludes corrective surgery and necessitates medical management [3,4,8]. In contrast, the left lung is perfused exclusively through multiple MAPCAs and bronchial collaterals, whose intrinsic high resistance is thought to protect the distal pulmonary vascular bed from the pressure and volume overload that otherwise drive Eisenmenger physiology, which acts as a protective mechanism in pulmonary atresia with VSD [3,4,8].

Definitive differentiation between elevated pulmonary vascular resistance and irreversible Eisenmenger physiology requires cardiac catheterization with vasoreactivity testing [8]; however, the patient declined catheterization, and true pulmonary vascular resistance and the reversibility of any pulmonary hypertension in the Hemitruncus-supplied right lung could therefore not be established in this case. The absence of peripheral pruning on CT and the predominance of increased, rather than reduced, broncho-vascular markings on chest radiography in the right lung are reassuring against advanced, irreversible pulmonary vascular disease at the time of imaging, but do not exclude an earlier or evolving stage of pulmonary vascular remodeling in the absence of invasive hemodynamic confirmation.

Despite uncorrected TOF, this patient had no growth retardation or history of cerebrovascular accidents. Compensatory polycythemia may have helped maintain systemic oxygen delivery, while MAPCA-mediated pulmonary blood flow likely prevented profound hypoxemia, reflecting physiological adaptation [8]. Hemoptysis in MAPCA-dependent CHD is typically caused by rupture of hypertrophied collateral vessels into the bronchial tree. Coagulopathy at presentation represented a major contraindication to immediate endovascular intervention. Cardiac CT played an important role in mapping the MAPCAs. Selective embolization using coils or vascular plugs of the targeted collaterals remains the preferred treatment [9]. 

This case poses a management challenge due to coagulopathy and anatomical complexity of MAPCA-dependent pulmonary blood flow, the severity of pulmonary vascular disease, which could not be characterized in the absence of cardiac catheterization. Definitive surgical treatment would require cardiac catheterization to assess pulmonary artery pressure, vascular resistance, and reversibility specifically, to determine whether the Hemitruncus-supplied right lung has developed fixed Eisenmenger physiology, which would preclude corrective surgery [9,10].

If feasible, unifocalization of MAPCAs, implantation of RPA to form an RPA-LPA confluence, and a modified BT shunt would be required, followed by intracardiac repair with VSD patch closure and RVOT reconstruction; however, the operative risk is substantial and should be considered only after a comprehensive hemodynamic assessment [10]. In this case, the patient refused invasive assessment and surgical management and was kept on conservative treatment.

## Conclusions

This case highlights the exceptionally rare coexistence of uncorrected TOF, right Hemitruncus, and MAPCA-dependent left pulmonary circulation in an adult survivor, posing management challenges. Cardiac CT was crucial for establishing the complete anatomical diagnosis, delineating MAPCAs, and guiding further management. Nevertheless, this case report underscores the role of cardiac CT in the accurate diagnosis of adult complex CHDs and treatment planning.
